# Cloning and Characterization of Porcine 4Ig-B7-H3: A Potent Inhibitor of Porcine T-Cell Activation

**DOI:** 10.1371/journal.pone.0021341

**Published:** 2011-06-27

**Authors:** Weiwei Chen, Zhibo Hou, Chunman Li, Sheng Xiong, Henggui Liu

**Affiliations:** 1 National Key Laboratory of Veterinary Biotechnology, Harbin Veterinary Research Institute of Chinese Academy of Agricultural Sciences, Harbin, China; 2 Treatment and Research Center for Infectious Diseases, Beijing 302 Hospital, Beijing, China; 3 Biomedical R&D Center, Guangdong Provincial Key Laboratory of Bioengineering Medicine, National Engineering Research Center of Genetic Medicine, Jinan University, Guangzhou, China; University Paris Sud, France

## Abstract

**Background:**

Members of the B7 superfamily costimulate the proliferation of lymphocytes during the initiation and maintenance of antigen-specific humoral and cell-mediated immune responses. B7-H3 (CD276) is a newly identified member of the B7 superfamily. It has been shown that B7-H3 plays a significant role in regulating T cell response in humans and mice, but it is not known whether a counterpart of human or murine B7-H3 exists in porcine species.

**Methodology/Principal Findings:**

We cloned the porcine *4ig-b7-h3* gene using a blast search at the NCBI database with human *b7-h3*, RT-PCR and 3′-terminus RACE. Protein sequence analysis showed that the protein encoded by this gene contained 4Ig-like domains and was 90.88% identical with human 4Ig-B7-H3. Results of Dot-blot hybridization and RT-PCR showed that B7-H3 was broadly distributed in porcine tissues mainly as two isoforms, 2Ig-B7-H3 and 4Ig-B7-H3, of which 4Ig-B7-H3 was dominant. We further demonstrated that porcine 4Ig-B7-H3 was able to inhibit the proliferation and cytokine production of porcine T cells activated through the TCR pathway, similar to human B7-H3.

**Conclusion:**

We cloned the porcine *4ig-b7-h3* gene and demonstrated that the porcine 4Ig-B7-H3 serves as a negative regulator for the T-cell immune response.

## Introduction

An optimal T cell response needs two signals. One is delivered through the antigen-specific T cell receptor (TCR), while the other is introduced by the costimulatory molecules that play either a stimulatory or inhibitory role. Members of the B7 protein superfamily act as costimulatory molecules regulating T cell responses after binding to their cognate counter-receptor. For example, during T cell activation, B7-1 and B7-2, two well-known members of the B7 superfamily, deliver positive costimulatory signals through CD28 to prevent tolerance and promote the growth and survival of T cells. However, binding of these two molecules by CTLA-4 will result in negative signaling to T cells [1]. Apart from B7-1 and B7-2, several new B7 superfamily members have been identified in recent years, such as B7h (B7-H2 or CD275), PD-L1 (B7-H1 or CD274), PD-L2 (B7-DC or CD273) and B7-H4, all of which are involved in regulating T cell functions [2], [3], [4], [5], [6], [7], [8], [9].

B7-H3 is a type I transmembrane protein that was identified as a member of the B7 superfamily [10], [11]. The expression level of this protein is relatively low, however, it is broadly detected in human and murine normal tissues at the RNA level [10], [11]. The B7-H3 expression can be induced on T cells, natural killer cells, and antigen-presenting cells (APCs) including DCs and macrophages [12], [13]. There are two isoforms of B7-H3 identified in humans, 2Ig-B7-H3 and 4Ig-B7-H3. 2Ig-B7-H3 contains a single Ig-V- and Ig-C-like domain, whereas 4Ig-B7-H3 has duplicated Ig-V- and Ig-C-like domains. Although their protein sequences differ, both isoforms show the similar functions [14]. At the protein level, a soluble isoform of human B7-H3 has also previously been identified [15]. Studies have shown that B7-H3 in humans and mice play important regulatory roles in T and NK cell responses [12], [16], [17]. However, the conclusions about its functions remain conflicting. The original research showed B7-H3 to play a stimulatory role through enhancement of T cell proliferation and cytokine production upon TCR activation [10], which was supported by an additional number of studies [18], [19], [20]. However, others presented evidences showing it to be, instead, a negative regulator of T cell response [12], [14], [16], [21], [22], [23]. The receptor of B7-H3 has not been identified. It is possible that these conflicting results in the function of B7-H3 might be related to its binding to different receptors.

A few members of the human B7 superfamily, including B7-1, B7-2, PD-1, PD-L1 and PD-L2, have porcine homologues with similar functions as their human counterparts [24], [25], [26], [27], [28], [29]. As a new important regulator of T cell response identified in both humans and mice, it is worthy to investigate if B7-H3 also exists in porcine and plays a similar role in the porcine T cell response. In this study, we cloned the *4ig-b7-h3* gene from porcine pulmonary alveolar macrophages (PAM), which showed 90.88% identity to the human 4Ig-B7-H3 Protein sequence. We then demonstrated that it was broadly distributed in porcine tissues and had inhibitory functions for the proliferation and cytokine production of porcine T cells.

## Results

### Cloning and characterization of the porcine *4ig-b7-h3* gene

**Figure 1:**
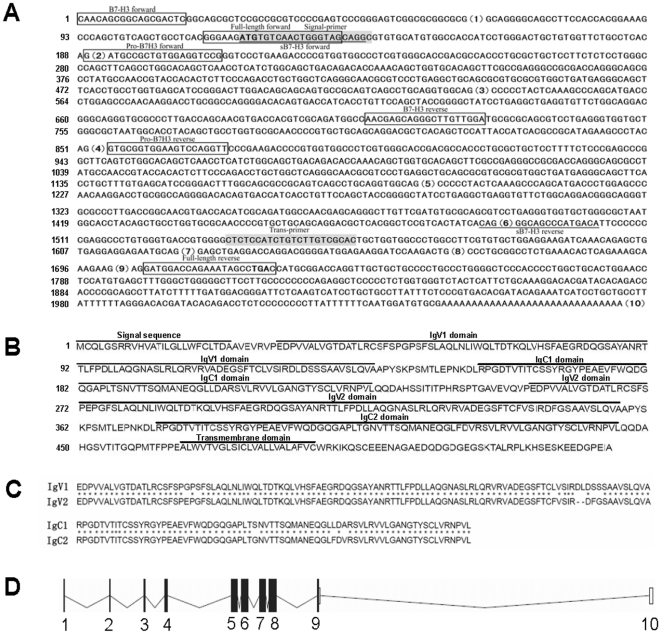
Analysis of cDNA and the protein sequence of porcine 4Ig-B7-H3. A, The cloned cDNA. The boxed, underlined and gray colored sequences show the positions of all used primers. The numbers in the brackets are the exon order of porcine B7-H3 in the genome. The strong ATG and TGA are initiation and stop codons. All exons contain the complete sequence in the genome but exon 1 in the cDNA. The numbers on the left are the base count of cDNA. B, The predicted protein sequence. The numbers on the left are the count of amino acids . All predicted domains are marked above the sequence. C, Alignment of the duplicated amino acid sequences of the two IgV and IgC-like domains, respectively. Asterisks indicate the identical amino acids. D, Schematic structure of CD276 nucleotide sequence in the porcine genome. The filled panes and lines represent Exons and Introns, respectively. The numbers under the schematics is the order of the Exons in the genome.

To clone the porcine *b7-h3* gene, a nucleotide BLAST search was carried out at the National Center for Biotechnology Information (NCBI) database using the human *b7-h3* gene. A homological Sus scrofa EST sequence (accession number: BP144068.1) was obtained. As the EST sequence does not contain a complete ORF, we first amplified the EST using a specific primer pair (B7-H3 forward and B7-H3 reverse in Figure 1A) designed according to the Sus scrofa EST sequence. After sequencing the obtained fragment, we further carried out a 3′-terminus RACE. As shown in Figure 1A, a total length of 2069-bp mRNA was obtained after the assembly of the EST and the fragment obtained by RACE. It contains a short 5′-untranslated stretch and 1605-bp complete ORF encoding 534 amino acids (Figure 1B) and a 345-bp 3′-untranslated sequence with a poly (A) tail. The deduced protein sequence consists of a short signal peptide and a duplicated peptide sequence with two pairs of IgV-IgC-like domains, followed by a transmembrane domain and a short cytoplasmic tail (Figure 1B). Thus, it belongs to the immunoglobulin superfamily. Further alignment of the deduced protein with human 4Ig-B7-H3 (accession number: NM_001024736), showed that they are highly homological in protein sequence reaching up to 90.88% identity. These results indicated that our cloned gene is the counterpart of human CD276 in porcine. Accordingly, we named it porcine 4Ig-B7-H3. A sequence search in the pig genome showed that the porcine *4ig-b7-h3* gene resides on chromosome 7 (from 65153991 to 65183019, accession number: LOC100153704) and contains 10 predicted exons (Figure 1A and D). Sequence alignment demonstrated a perfect match between the ORF of the cloned porcine *4ig-b7-h3* and the predicted exons in the genome.

### The B7-H3 is broadly distributed as a 4Ig-B7-H3 isoform in porcine

**Figure 2:**
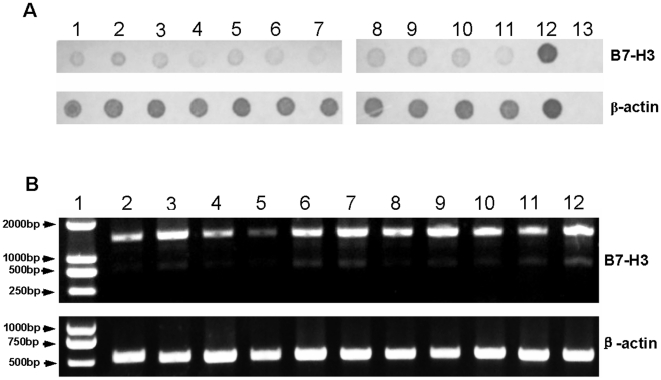
Distribution and analysis of the porcine B7-H3. A, Dot-blot hybridization assay of mRNA from different tissues using the probes of 4Ig-B7-H3. Samples of PBMCs, PAM, heart, liver, spleen, lung, kidney, brain, lymph nodes, small intestine, tonsil were loaded from dot 1 to 11, respectively, with positive control (dot 12) and negative control (dot 13). The upper and lower panels were B7-H3 and β-actin, respectively. B, Analysis of the porcine B7-H3 isoforms. The normalized PCR products of PBMCs, PAM, heart, liver, spleen, lung, kidney, brain, lymph nodes, small intestine, tonsil were loaded from lane 2 to 12 with DNA ladder marker (lane 1). The upper and lower panels were the PCR products of B7-H3 and β-actin, respectively.

To investigate the tissue distribution of porcine B7-H3, a dot-blot hybridization assay was performed. Total mRNA were extracted from different porcine tissues, including PBMCs, PAM, heart, liver, spleen, lung, kidney, brain, lymph nodes, small intestine and tonsil. The obtained mRNAs were probed using Digoxigenin Labeled PCR products of porcine 4Ig-B7-H3. As shown in Figure 2A, all tested tissues expressed B7-H3. There are two forms of human B7-H3, 2Ig-B7-H3 and 4Ig-B7-H3 [10], [17]. Because porcine 4Ig-B7-H3 contains repeat sequences of IgV-C-like domain, the probe generated from porcine 4Ig-B7-H3 in the dot-blot hybridization could not rule out the existence of 2Ig-B7-H3. To investigate whether 2Ig-B7-H3 exists in porcine tissues, we designed a specific primer pair with the forward primer located in the signal-encoding region and the reverse primer in the transmembrane domain. The amplified products from 4Ig-B7-H3 should be 1438 bp, but about 700 bp are from 2Ig-B7-H3 according to the analysis of the protein sequence of porcine 4Ig-B7-H3. As shown in Figure 2B, the dominant band amplified from the cDNA of all tissues was 4Ig-B7-H3, which was located between the 1 Kb and 2 Kb bands of the DNA ladder marker. There was also a small sized band in the PCR products of almost all cDNAs residing between 750 bp and 1 Kb, which was confirmed to be the *2ig-b7-h3* gene by sequencing. Further cloning the full length cDNA and analyzing the nucleotides of *2ig-b7-h3* showed porcine *2ig-b7-h3* contains eight exons (Exon 1, 2, 3, 6, 7, 8, 9 and 10) and encodes IgV2 and IgC2-like domains. These results revealed the broad distribution of B7-H3 in porcine tissues and similar to human B7-H3, the existence of two isoforms in porcine B7-H3, 2Ig-B7-H3 and 4Ig-B7-H3, of which 4Ig-B7-H3 is remarkably dominant, especially in PBMCs, brain and tonsil. Besides the transmembrane isoforms of B7-H3, there also exists a soluble form of human B7-H3 [15]. When we amplified the extracellular domain of porcine CD276 (Figure 2B), a medium sized band appeared between *4ig-b7-h3* and *2ig-b7-h3*. To address whether this band was the soluble form of porcine CD276, we cloned and sequenced its full length cDNA with the primer pair full-length forward and full-length reverse (Figure 1A). However, instead of getting the soluble form of porcine CD276, we found two additional isoforms of porcine CD276 with one isoform having the Exon 6 spliced out and the other one possessing a deletion of the first 216-bp nucleotides at Exon 3.

### PD-1 is not the receptor for porcine B7-H3

**Figure 3:**
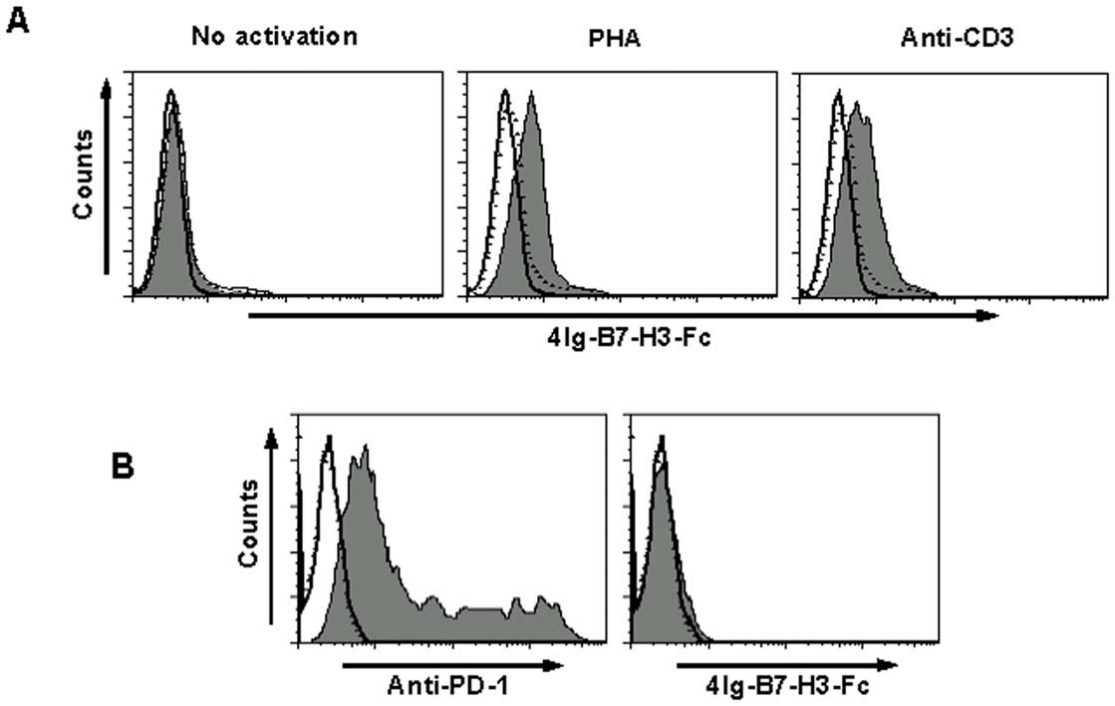
Analysis of a putative receptor of porcine 4Ig-B7-H3. A, Binding of porcine 4Ig-B7-H3-Fc to T cells. T cells treated in different conditions were stained with a combination of anti-CD3 mAb with Fc (solid line histogram) or isotypic Ab (dotted line histogram) or 4Ig-B7-H3-Fc (filled histogram). Left plot: T cells in PBMC were not activated. Middle plot: T cells in primary PBMCs were stimulated with 5 µg/ml of PHA for 24 h. Right plot: T cells in primary PBMCs were stimulated with 1 µg/ml of anti-porcine CD3 mAb coated on the 96-well plate for 48 h. B, PD-1 gene was transfected into 293 T cells. PD-1 expressed on the 293 T cells was stained with anti-PD-1 mAb (left, filled histogram) or 4Ig-B7-H3-Fc protein (right, filled histogram) or Fc (solid line histogram) or isotypic Ab (dotted line histogram) 24 hours later.

The receptor of B7-H3 has not been identified until now. Previously, it was shown that in humans, its putative receptor could be positively detected by the soluble B7-H3 protein on activated T cells [10]. Thus, it would be interesting to determine whether the putative receptor of porcine 4Ig-B7-H3 is also expressed on porcine T cells. To address this issue, we isolated the porcine PBMCs from big white pigs. The T cells in PBMCs were stimulated with either 5 µg/ml PHA or immobilized anti-porcine CD3 mAb. As shown in Figure 3A, when T cells were activated by PHA or anti-CD3, a positive staining was observed by FACS, indicating the binding of 4Ig-B7-H3 to T cells (the middle and right plots in Figure 3A). Previous reports showed PD-1 was up-regulated on activated T cells [30]. To investigate if PD-1 could be the potential receptor for porcine 4Ig-B7-H3, porcine PD-1 was transfected into 293T cells and stained with 4Ig-B7-H3-Fc. As shown in Figure 3B, although PD-1 was successfully expressed on 293T cells (left plot in Figure 3B), there was no observed positive staining by porcine 4Ig-B7-H3-Fc (right plot in Figure 3B), indicating that PD-1 is not the potential receptor. Taken together, these results implied that the porcine B7-H3 receptor is regularly expressed on activated T cells. Although it remains unknown which protein serves as the receptor for porcine B7-H3, our results have revealed that it may be a new protein different from PD-1.

### 4Ig-B7-H3 inhibits T cell functions in porcine

**Figure 4:**
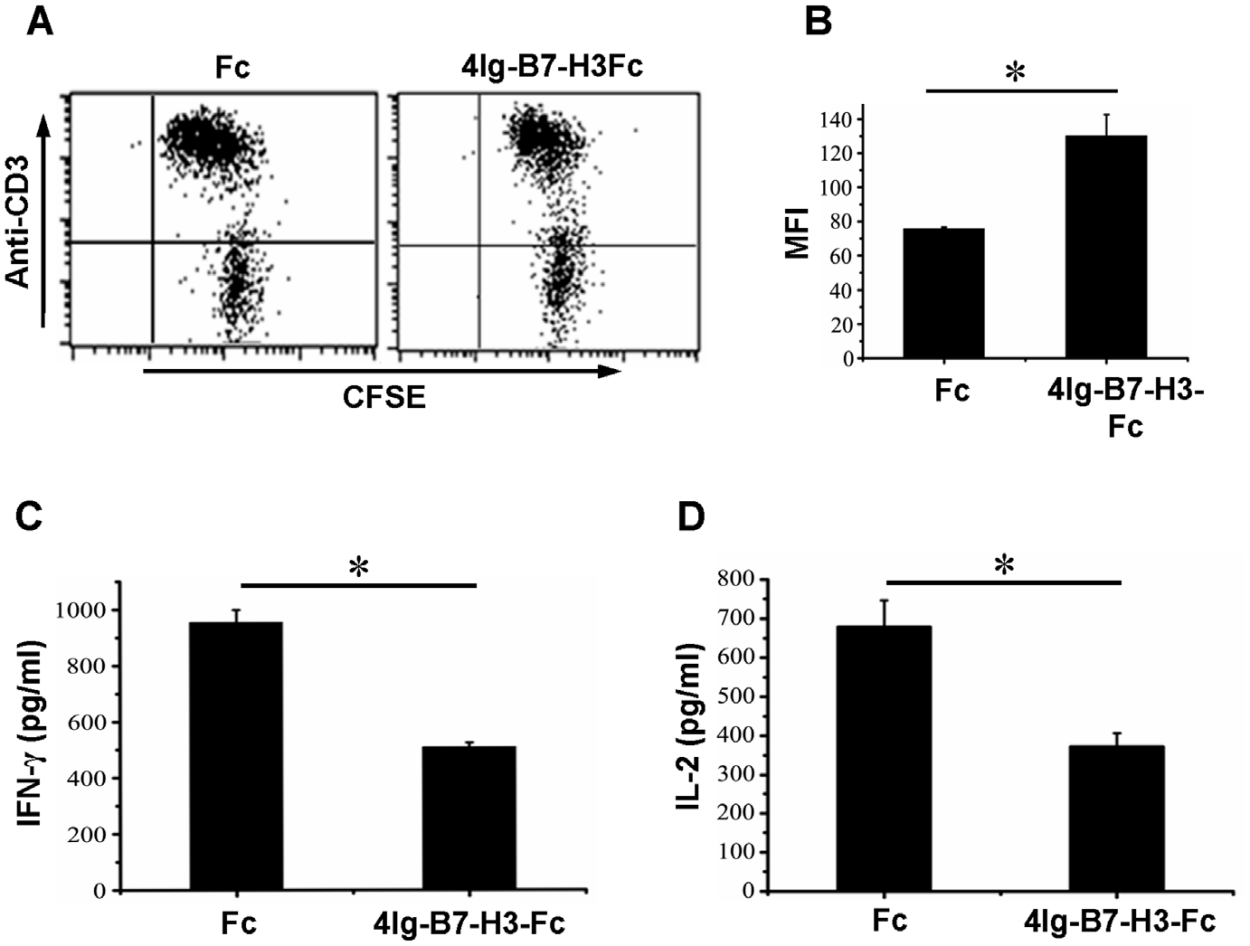
Porcine 4Ig-B7-H3 inhibited T cell functions. A, Freshly isolated porcine PBMCs were seeded to 96-well plate precoated with 1 µg/ml of anti-CD3 mAb plus either 10 µg/ml of 4Ig-B7-H3-Fc or Fc in 50 µl over night at 4°C. After culturing for 72 hours, T cell proliferation in the presence of Fc (left plot) or 4Ig-B7-H3-Fc (right plot) was analyzed. The plots were from one representative experiment of three independent samples. B, Statistic analysis of mean fluorescence intensity (MFI) on T cells (mean ± s.e.m). C and D, T cells purified from porcine PBMCs were seeded to the 96-well flat-bottomed plate precoated with 1 µg/ml of anti-CD3 mAb plus either 10 µg/ml 4Ig-B7-H3-Fc or Fc in 50 µl over night at 4°C. After culturing for 72 hours, supernatants were harvested to quantify IFN-γ (C) and IL-2 (D) concentration using ELISA kit. All data were from a representative of three independent experiments. _*_ means P<0.05.

To explore the functions of 4Ig-B7-H3 in porcine immune response, we first assessed the effect of porcine 4Ig-B7-H3 on T cell proliferation as done previously in the human B7-H3 functional assay [13]. As shown in Figure 4A and B, when the T cells activated by anti-porcine CD3 mAb were co-cultured with Fc or 4Ig-B7-H3 protein for 72 h, MFI of T cells in the Fc control group was significantly decreased, down to about 41.9% compared to the 4Ig-B7-H3 group, This indicated that the T cell proliferation in the 4Ig-B7-H3 group was significantly inhibited compared to the Fc control group. We further evaluated the effect of 4Ig-B7-H3 on the cytokine production of porcine T cells. Purified T cells were stimulated with anti-CD3 mAb in the presence of either 4Ig-B7-H3-Fc or Fc. The supernatants were harvested to quantify the expression of IFN-γ and IL-2 with ELISA kit after 72 hours. As shown in Figure 4C and 4D, the production of IFN-γ and IL-2 was significantly reduced in the presence of 4Ig-B7-H3-Fc, compared to the Fc control group, with inhibition up to 45% and 46.6%, respectively. Altogether, these results demonstrated that porcine 4Ig-B7-H3 plays a negative regulatory role on the porcine T cell functions.

### The expression of porcine B7-H3 is up-regulated on activated monocytes</p>

**Figure 5:**
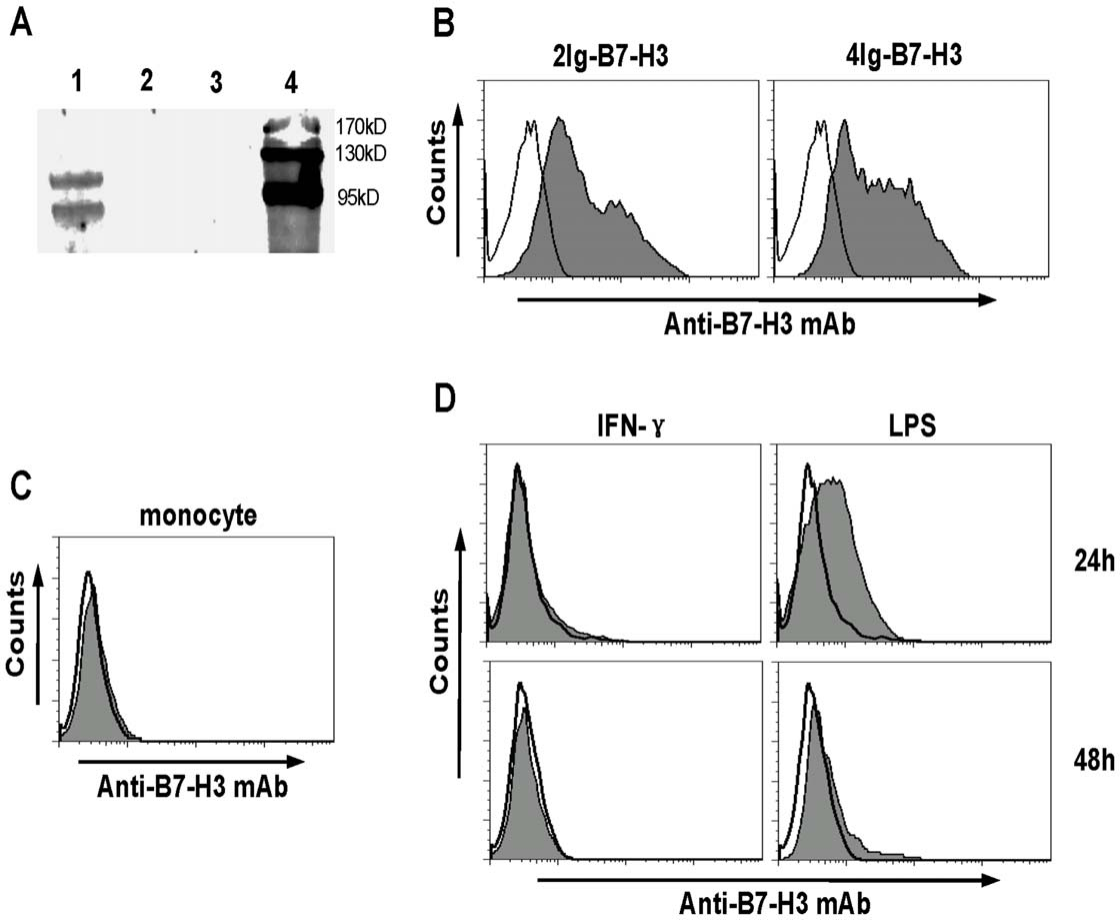
Analysis of porcine B7-H3 expression on monocytes. A, Immunoprecipitation assay. The membrane protein extracted from the porcine 4Ig-B7-H3-transfected 293 T cells (Lane 1) or vacant plasmid-transfected 293 T cells (Lane 2) was pulled down with anti-porcine B7-H3 mAb-bound protein-G agarose. The pulled-down proteins were submitted to western-blot assay. Lane 3: anti-porcine B7-H3 mAb-bound protein-G agarose control. Lane 4: protein marker. B, The analysis of the porcine 2Ig-(left plot) and 4Ig-B7-H3 (right plot) expressed on 293 T cells stained by the anti-porcine B7-H3 mAb (filled histogram) or isotypic Ab (open histogram). C, non-activated monocytes were stained with a combination of anti-CD14 with either anti-B7-H3 mAb (filled histogram) or isotypic Ab (open histogram). D, The monocytes were co-cultured with IFN-γ in a final concentration of 30 ng/ml (left plots) or LPS at a final concentration of 1 µg/ml (right plots) for 24 h (upper plots) or 48 h plots (lower plots). On the indicated time points, the monocytes were harvested to be stained with a combination of anti-CD14 with either anti-B7-H3 mAb (filled histogram) or isotypic Ab (open histogram).

Monocytes are a subgroup of immune cells that can interact with T cells and play a pivotal role in the T cell functions. As porcine 4Ig-B7-H3 was found to negatively regulate T cell function, we then wondered whether it was expressed on monocytes. To address this issue, we first produced the anti-B7-H3 mAb using a part of the ectodomain of 4Ig-B7-H3 to immunize mice. To validate the specificity of the anti-porcine B7-H3 mAb, we performed an immunoprecipitation assay. As shown in Figure 5A, when the membrane proteins purified from the 293 T cells transfected with porcine 4Ig-B7-H3 or vacant plasmid were pulled down with the anti-porcine B7-H3 mAb-bound protein-G agarose, only the 4Ig-B7-H3 protein was pulled down from the 4Ig-B7-H3-transfected 293 T cells (lane 1 in Figure 5A), There was no band for either the vacant plasmid-transfected 293 T cells (lane 2 in Figure 5A) or the mAb-bound protein-G agarose control (lane 3 in Figure 5A). This result showed the perfect specificity of mAb against porcine 4Ig-B7-H3. The anti-porcine B7-H3 mAb was able to specifically recognize both porcine 2Ig- and 4Ig-B7-H3 expressed on the cell membrane (Figure 5B). The mAb was used to test the expression level of B7-H3 on freshly isolated or activated porcine monocytes. As shown in Figure 5C, porcine B7-H3 was undetectable on the freshly isolated monocytes (Figure 5C). We further examined the porcine B7-H3 expression on monocytes activated with LPS or IFN-γ for 24 h and 48 h. As shown in the right plots of Figure 5D, the porcine B7-H3 was up-regulated on monocytes activated by LPS for 24 h, but its expression was then eliminated to undetectable levels at 48 h. However, IFN-γ could not up-regulate the expression of the porcine B7-H3 under the same conditions (left plots in Figure 5D). These results indicated that the expression of porcine B7-H3 on monocytes can be up-regulated in certain circumstances.

## Discussion

An optimal T cell response depends on a combination of signals delivered through the antigen-specific T cell receptor (TCR) and accessory signals. The B7 superfamily proteins provide stimulatory or inhibitory accessory signals for T cell responses. In this report, we cloned and identified the porcine 4Ig-B7-H3, a new member of the B7 superfamily. It contains four immunoglobulin-like domains, which has high sequence homology with the corresponding human 4Ig-B7-H3 [13], [17], [31]. Human 4Ig-B7-H3 is generated from the double copies of IgV-C-like domains in the genome [14], and the porcine 4Ig-B7-H3 has a similar organization. Both human and porcine CD276 have two isoforms, 2Ig-B7-H3 and 4Ig-B7-H3 [13], [31]. Porcine 4Ig- and 2Ig-B7-H3 isoforms are broadly expressed in all porcine tissues with 4Ig-B7-H3 as the dominant form (Figure 2). These results indicated that the expression profiles of porcine and human CD276 are similar and that the members of the B7-superfamily are highly conserved between human and porcine species [25], [27], [29], [32]. In contrast to human CD276, the soluble form of porcine CD276 was not detected. Instead, two other isoforms of CD276 were identified in porcine tissues. It is noteworthy that in contrast to human B7-H3, the porcine B7-H3 mRNA was detected in the PBMCs [10]. Recently, a porcine CD276 sequence had been deposited in the GenBank (Assession number: XM_001925645) that encodes a 552 amino acid protein. It possesses a longer intracellular domain than the CD276 protein reported here. We have tried to look for this longer B7-H3 in our samples by sequencing more clones, but failed to find it. Because the XM_001925645 sequence was predicted by automated computational analysis according to the genomic sequence, it probably does not exist in porcine tissues in view of our results and the protein sequence of human CD276. There are about 39 species of CD276 sequences deposited in the Ensemb database so far, most of which are predicted by automated computational analysis. These predicted CD276 sequences contain from 4 to 21 exons. Analysis of the protein sequences encoded by these predicted CD276 showed that some only encode 2Ig-B7-H3, such as Felis catus (ENSFCAG00000006344) and Gallus gallus (ENSGALG00000001729) and others only encode 4Ig-B7-H3, such as Gorilla gorilla (ENSGGOG00000013930), pongo pygmaeus (ENSPPYG00000006626), Pteropus vampyrus (ENSPVAG00000015344) and Procavia capensis (ENSPCAG00000001931). There also are species that encode both 4Ig- and 2Ig-B7-H3, such as Loxodonta africana (ENSLAFG00000014932), macaca mulatta (ENSMMUG00000005448) and Pan troglodytes (ENSPTRG00000007261).

Human B7-H3 was first described as a potent co-stimulatory molecule, stimulating T cells to proliferate and secrete IFN-γ [10], [18], [19]. Other studies showed that human and murine B7-H3 had inhibitory functions [12], [14], [16]. This discrepancy is perhaps due to the existence of multiple receptors of B7-H3 on human and murine T cells or to different experimental conditions that lead to the engagement of a stimulatory or an inhibitory receptor on T cells. Our study found that porcine 4Ig-B7-H3 inhibited both T cell proliferation and cytokine production (Figure 4), supporting B7-H3 as an inhibitory molecule in the T cell responses. In our results, the median inhibition is less than 50% for porcine 4Ig-B7-H3 compared to 74% for human B7-H3 [21]. The lower inhibitory efficiency of the porcine B7-H3 compared to the human B7-H3 perhaps results from the different methods we used. The radioactive assay with Methyl-^3^[H]-thymidine used for the human B7-H3 experiments is more sensitive than the CFSE dilution assay used in our experiment. Although both porcine and human B7-H3 inhibit the secretion of IFN-γ and IL-2, the inhibitory efficiency is different: the inhibition of IFN-γ and IL-2 secreted by porcine B7-H3 is 45% and 46.6% (Figure 4C and D) respectively, compared to over 50% by human B7-H3 [21].

The predicted molecular weight of porcine 4Ig-B7-H3 is about 57 kD according to the protein sequence. However, the pulled-down 4Ig-B7-H3 showed two bands, one was about 95 kD and the other was between 95 kD and 130 kD (Figure 5A). These results indicated that porcine B7-H3 is highly modified in mammalian cells and that there are two kinds of modifications. In humans and mice, B7-H3 was up-regulated on DCs by some factors [10], [12]. Given that DCs are important antigen presenting cells (APCs), the up-regulation of B7-H3 on DCs has a significant role in the immune response [12]. However, we were unable to test this effect because the commercial porcine cytokines were not available to produce porcine DCs. Considering that monocytes are important precursors of DCs, we analyzed the B7-H3 expression on porcine monocytes. Our results demonstrated that LPS was able to stimulate B7-H3 expression on porcine monocytes, which is similar to the result with human monocytes [33]. On the other hand, IFN-γ enhances B7-H3 expression on mouse DCs [12], but has no effect on porcine monocytes (Figure 5D), indicating the variation in the different species.

The interaction between the B7 and CD28 families provides an important accessory signal for T cell functions. The expression variation of these molecules on immune cells plays a role on the progression of infectious diseases and cancers. HIV causes chronic infection in part because it enhances PD-1 expression [34]. Over-expression of PD-L1 on cancer cells promotes cancer progression [35], [36]. Up-regulation of PD-L1 correlates with the disease progression of HIV-1 infections [37]. Most available data show that the change in human B7-H3 expression affects tumor progression [38], [39], [40], [41]. There are viruses that infect PAM and cause chronic infection in porcine species, such as porcine reproductive and respiratory syndrome virus (PRRSV) [42], [43]. However, the mechanism of persistent infection of PRRSV remains unknown. Porcine B7-H3 is expressed on the porcine PAM (Figure 2). Considering the negative regulatory role of porcine B7-H3 in immune responses, it is conceivable that viruses, such as PRRSV, may exploit the B7-H3/receptor pathway to invade porcine immune system and cause persistent infection. Further investigation would be needed to address this hypothesis in the pig model.

## Materials and Methods

### Antibodies, reagents and animals

PE-Goat anti-mouse IgG Ab, FITC-Goat-anti-rabbit IgG Ab (ZSGB-BIO), FITC-anti-pig-CD14 mAb (AbD Serotech), PE-anti-pig CD3 (Southern Biotech) and purified anti-pig CD3 mAb (Southern Biotech) were available from companies. Recombinant pig IFN-γ was purchased from Geneway (GenWay Biotech, Inc.). PHA, CFSE, and protein G agarose were obtained from Sigma-Ardrich Inc. The protein G magnetic beads were bought from Millipore. The DIG High Prime DNA Labeling and Detection Starter Kit were purchased from Roche. 2–3 months old of big white pig and 6–8 weeks old of BALB/c mouse were used in this research. Mouse and pig care and breeding were performed according to the guidelines and approved by the Animal Care and Use Committee of the Jinan University under the permit number SCXK (Guangdong) 2003–0002.

### Pulmonary alveolar machrophages Isolation and gene cloning

Porcine pulmonary alveolar machrophages (PAM) were isolated according to the previous report [44]. The total RNA of PAM was isolated and the cDNA was produced using RNeasy® plus mini kit (QIAGEN) and PrimeScript® 1^st^ strand cDNA synthesis kit (TaKaRa), respectively. To clone the porcine B7-H3, a blast search was carried out in the EST database with human B7-H3 gene. According to the obtained sequence, a specific primer pair, B7-H3 forward 5′-CAACAGCGGCAGCGACTC-3′ and B7-H3 reverse 5′-TCCAACAAGCCCTGCTCGTT-3′, was designed. PCR was performed under the following conditions: denature at 95°C for 5 min, followed by 35 cycles at 94°C for 30 sec, annealing at 60°C for 30 sec and extension at 72°C for 1 min. The PCR products were inserted into the pMD18-T vector and sequenced. The 3′-full RACE was carried out using the Kit (TaKaRa). The full-length of ORF of porcine B7-H3 was amplified using the following primer pair: Full-length forward 5′- GGGAAGATGTGTCAACTGGGTAG-3′ and Full-length reverse 5′- GTCAGGCTATTTCTGGTCCATC -3′. The amplified full-length of porcine B7-H3 was inserted into pcDNA3.1(+) vector to generate pcB7-H3 recombinant vector.

### Generation of Ig-fusion proteins

The ectodomain of the porcine 4Ig-B7-H3 was amplified using the following primers: sB7-H3 forward: 5′-ATGTGTCAACTGGGTAGCAGG-3′ and sB7-H3 reverse: 5′- TGTCATGGGCTGCCCTG-3′. The amplified ectodomain of porcine 4Ig-B7-H3 was fused with rabbit Fc in one ORF and inserted into pcDNA3.1(+) vector to generate pcB7-H3-Fc. The soluble protein, 4Ig-B7-H3-Fc, was produced through transfecting pcB7-H3-Fc to 293 T cells and purified as previously reported [29], [45]. The concentration of the purified 4Ig-B7-H3-Fc was determined with the rabbit IgG ELISA quantification kit (Bethyl).

### Monocytes isolation

Porcine PBMCs were first isolated from freshly-drawn blood by Ficoll-Paque density gradient centrifugation according to the previous report [29]. Primary blood monocytes (PBM) were isolated as reported in humans [46]. PBM were resuspended in RPMI 1640 medium supplemented with 5% autologous plasma and cultured for 1 h. Following removal of non-adherent cells, adherent cells have been shown to be >90% positive using anti-CD14 antibodies staining by flow cytometer.

### Immunization, monoclonal antibody (mAb) production and FACS analysis

To produce mAb specific against porcine 4Ig-B7-H3, a part of the ectodomain was amplified using the following primers: pro-B7H3 forward 5′-GATGCCGCTGTGGAGGTCCG-3′ and pro-B7H3 reverse 5′-TCAAACCTGGACTTCCACCGCAC-3′. The fragment was inserted into pGEX-6p-1 vector and expressed in the bacteria. The GST-B7-H3 fusion protein was purified with Glutathione-agarose (Sigma-Ardrich, Inc.). The protein purity was confirmed using SDS-PAGE assay. The GST-B7-H3 was used to immunize 6-8 weeks old of mice with 100 µg/mouse in the complete or incomplete Freund's adjuvant. After immunization for three times with an interval of 2 weeks, the mice were sacrificed to isolate splenocytes for cell fusion with Sp2/0 cell line. The positive clone was screened with GST-B7-H3 protein by ELISA assay and 4Ig-B7-H3-transfected 293 T cell line by immunofluorescence assay. To analyze B7-H3 expression on monocytes with anti-B7-H3 mAb, the cells first stained with anti-B7-H3 mAb and PE-anti-mouse IgG Ab. After washing twice, the cells were stained with FITC anti-CD14 mAb. The stained cells were analyzed in FACSCalibar and FlowJo software.

### Immunoprecipitation assay

To perform the immunoprecipitation assay, the membrane proteins were first extracted using Mem-PER eukaryotic membrane protein extraction reagent kit (Thermo). The anti-porcine B7-H3 mAb was first incubated with protein-G agarose for 1 h. After washing three times with PBS, the anti-porcine B7-H3 mAb-bound protein-G agarose was added to the purified membrane proteins and incubated at 4°C overnight. The protein-G agarose was washed three times with PBS after incubation and submitted to western-blot assay. The porcine B7-H3 was probed with the anti-porcine B7-H3 mAb and IRDye 680 donkey anti-mouse IgG (LI-COR Biesciences) and scaned with Li-Cor.

### Dot-blot hybridization

The dot-blot hybridization assay was carried out as previously reported [47]. Briefly, mRNA was purified from the total RNA of the different porcine tissues using FastTrack MAG mRNA isolation kit (Invitrogen). 1 µg mRNA was dropped to HybondN nylon membrane (Amersham International) and hybridized with the digoxigenin-labeled PCR products of the porcine B7-H3 amplified with B7-H3 forward and reverse primer pair (5′-CAACAGCGGCAGCGACTC-3′and 5′-TCCAACAAGCCCTGCTCGTT-3′) in DIG Easy Hyb solution (Roche) at 55°C overnight. The blot was washed twice with 2 x SSC containing 0.1%SDS at room temperature followed twice with 0.1 x SSC containing 0.1%SDS at 55°C. The signals were analyzed according to the manufacturer's instruction. The porcine β-actin was probed as was done in the porcine B7-H3 assay to serve as an internal reference. The probe of the porcine β-actin was produced with the primers 5′-CATGAAGATCCTCAC-3′ and 5′-TGATCCACATCTGCT-3′. The mRNA extracted from 293 T cells transfected with the porcine 4Ig-B7-H3 or porcine β-actin served as the positive control.

### RT-PCR analysis of porcine B7-H3 mRNA

To identify the isoforms of the porcine B7-H3, total RNA was prepared from different tissues using Trizol (Invitrogen), and cDNA was produced with the Kit according to the manufacturer's protocols. cDNA was amplified using Signal-primer 5′-ATGTGTCAACTGGGTAGCAGGC-3′ and Trans-primer 5′-GTGCGACAAGACAGATGGAGAG-3, which resides in the signal and transmembrane regions, respectively. The β-actin was amplified with the primers mentioned above as internal reference. The PCR products of the porcine B7-H3 and β-actin were first normalized with the equal number of added templates, then a total of 6 ul products were subjected to analysis in 1% agarose gel.

### T cell proliferation and cytokine assay

Porcine PBMCs were isolated using Ficoll-Hypaque gradient centrifugation method and labeled with 2.5 µM CFSE as already published [29]. 5×10^5^ cells/well PBMCs labeled with CFSE were seeded to 96-well flat-bottomed plate precoated with 1 µg/ml anti-CD3 plus either 10 µg/ml porcine 4Ig-B7-H3-Fc or Fc, as the previous reports [18], [29]. After culturing for 72 h, the T cell proliferation was analyzed based on CFSE dilution by FACS assay. To analyze the effect of 4Ig-B7-H3 on the cytokine production of porcine T cells, T cells were purified from freshly isolated porcine PBMCs using protein G Magnetic beads. Briefly, T cells in PBMCs were first stained with anti-CD3 mAb for 10 min at 4°C. After washing twice, the equal number of magnetic beads were added and co-cultured for 15 min at 4°C. The T cells bound to magnetic beads were isolated with the magnet and released through culture for 2 hours at 37°C. The purified T cells were seeded to the wells of the plate precoated with 1 µg/ml anti-CD3 plus either 10 µg/ml porcine 4Ig-B7-H3-Fc or Fc. The culture supernatants were collected at 72 h and the concentration of cytokines was determined by sandwich ELISA Kits (Invitrogen) according to the manufacturer's instructions.

### Statistical analysis

Statistical analysis of the T cell proliferation and cytokine assay were performed using the Student's *t* test for paired variables. Statistical differences were considered when *p*<0.05.
